# Synthesis of hybrid hydrazino peptides: protected vs unprotected chiral α-hydrazino acids

**DOI:** 10.1186/s40064-015-1288-9

**Published:** 2015-09-17

**Authors:** Josipa Suć, Ivanka Jerić

**Affiliations:** Division of Organic Chemistry and Biochemistry, Ruđer Bošković Institute, Bijenička cesta 54, 10000 Zagreb, Croatia

**Keywords:** Acylation, Amino acids, Peptidomimetics, Synthetic methods

## Abstract

Peptidomimetics based on hydrazino derivatives of α-amino acids represent an important class of peptidic foldamers with promising biological activities, like protease inhibition and antimicrobial activity. However, the lack of straightforward method for the synthesis of optically pure hydrazino acids and efficient incorporation of hydrazino building blocks into peptide sequence hamper wider exploitation of hydrazino peptidomimetics. Here we described the utility of *N*^α^-benzyl protected and unprotected hydrazino derivatives of natural α-amino acids in synthesis of peptidomimetics. While incorporation of *N*^α^-benzyl-hydrazino acids into peptide chain and deprotection of benzyl moiety proceeded with difficulties, unprotected hydrazino acids allowed fast and simple construction of hybrid peptidomimetics.

**Electronic supplementary material:**

The online version of this article (doi:10.1186/s40064-015-1288-9) contains supplementary material, which is available to authorized users.

## Background

Proteins and their conjugates are key players in fundamental molecular functions that define life as we know. Despite the relatively small number of constitutive elements, 20 canonical amino acids, the structural and functional diversity of proteins stems from their ability to adopt discrete three-dimensional folded structures. Understanding the interplay between amino acid sequence, protein structure and a biological function remains one of the most challenging tasks (Horne 2011). Traditionally, peptides and proteins were placed far away from the focus of medicinal chemistry owing to the poor enzymatic stability and cell permeability. However, peptide-based therapeutics became highly important over the last few decades. The increased number of identified therapeutic targets and substantially upgraded delivery systems made peptide market growing almost twice as fast as the overall pharmaceutical market (Kaspar and Reichert 2013). Attempts to mimic folding properties of proteins led to the development of “foldamers”, non-natural oligomers able to adopt stable three-dimensional structure (Gellman 1998; Hill et al. 2001; Bautista et al. 2007).

Research efforts in construction of foldamers with predictable folding properties and desirable biological responses are directed mainly toward two classes of oligomers: arylamide and peptidic foldamers. Arylamide foldamers are composed of either aryl amino acids or combinations of aryldiamines and aryldiacids, where intramolecular hydrogen bonding and the intrinsic rigidity of arylamide units dictate conformation (Li et al. 2008; Zhu et al. 2011; Kudo et al. 2013; Guichard and Huc 2011). Peptidic foldamers are formally derived from the natural α-peptide sequence via backbone homologation (Horne 2011; Martinek and Fülöp 2012; Bandyopadhyay et al. 2014; Hegedüs et al. 2013; Avan et al. 2014). Intervention into peptide backbone by incorporation of non-natural amino acids or replacement of peptide bond with isosteres has a major implication on peptidomimetic properties. Medium-sized bridged heterocycles (La-Venia et al. 2014), sulfono-γ-amino acids (Wu et al. 2015), cyclopentane-based γ-amino acid (Giuliano et al. 2013), 2-aminobenzenesulfonic acid (Kale et al. 2013), *N*-amino-imidazolin-2-ones (Proulx and Lubell 2012) are some examples of effective secondary structure inducers upon incorporation into peptide backbone. Replacement of α-carbon or backbone extension leads to oligomers with well-established folding properties: azapeptides, azadepsipeptides, β- and γ-peptides, aminoxypeptides, and hydrazinopeptides (Avan et al. 2014). Hydrazino derivatives of α-amino acids can be derived from β-amino acids through replacement of β carbon atom with nitrogen. Repulsion of lone electron pairs placed at neighbouring nitrogen atoms, yields the observed pronounced rigidity of peptidomimetics with incorporated hydrazino derivatives (Cheguillaume et al. 2001). Also, intramolecular hydrogen bonding pattern in such peptidomimetics promotes formation of unique secondary structure, known as hydrazino-turn (Acherar et al. 2013; Salaün et al. 2006; Cheguillaume et al. 2001). Hydrazino-based peptidomimetics show promising biological activities, like protease inhibition (Bordessa et al. 2013; Aubin et al. 2005) and antimicrobial activity (Laurencin et al. 2012a, b). It is therefore important to have a method for fast and easy construction of such templates.

The most utilized hydrazino building block described in the literature is *N*-substituted hydrazino acetic acid; an achiral monomer readily prepared from substituted hydrazines and bromoacetic esters (Bonnet et al. 2003). Synthesis of optically pure hydrazino derivatives is a more challenging task. Generally, there are three approaches (Avan et al. 2014; Maraud and Vanderesse 2004): *N*-amination of *N*-benzyl derivatives of natural amino acids with *N*-Boc oxaziridines; Shestakov rearrangement, the conversion of urea derivatives to hydrazines using hypochlorite; and the conversion of α-amino acids to α-bromo acids followed by the nucleophilic substitution reaction of hydrazine with inversion of configuration. There are many drawbacks associated with each procedure. Electrophilic amination with *N*-Boc oxaziridines ensures optical integrity of the obtained hydrazino acids, however procedure suffers from the lack of reproducibility. Nucleophilic substitution of α-bromo acids with hydrazine is a rather simple and economic two-step procedure, but incomplete conversions were observed in some cases (Panda et al. 2013). Also, the adjacent nitrogen atoms, Nα and Nβ are both reactive; therefore regioselectivity during the synthesis of hydrazino-based peptidomimetics arose as a central issue when using derivatives with unprotected Nα atom (Maraud and Vanderesse 2004).

**Scheme 1:**
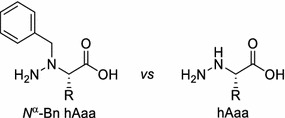
Structure of *N* ^α^-benzyl hydrazino acid (*N* ^α^-Bn hAaa) and hydrazino acid (hAaa)

We present here our findings on synthesis of di- and tripeptides with embedded hydrazino acids derived from natural amino acids. Both, *N*^α^-benzyl hydrazino acids (*N*^α^-Bn hAaa) and unprotected hydrazino acids (hAaa) (Scheme 1) were used to test their utility in synthesis of hybrid peptidomimetics.

## Experimental section

Reactions were monitored by TLC on Silica Gel 60 F254 plates (Merck) using detection with ninhydrin. The melting points were determined on a Tottoli (Büchi) apparatus and were uncorrected. Column chromatography was performed on Silica Gel (Merck, 0.040–0.063). Chiral TLC was performed on chiral Silica Gel 60 F254 plates (Aldrich). NMR spectra were recorded on 600 and 300 MHz Bruker spectrometers, operating at 150. 92 or 75.47 MHz for ^13^C and 600.13 or 300.13 MHz for ^1^H nuclei. TMS was used as an internal standard. HRMS analysis was performed on MALDI-TOF/TOF mass spectrometer operating in reflectron mode. Mass spectra were acquired by accumulating three spectra after 400 laser shots per spectrum. Calibrant and analyte spectra were obtained in positive ion mode. Calibration type was internal with calibrants produced by matrix ionization (monomeric, dimeric and trimeric CHCA), with azithromycin and angiotensin II dissolved in a-cyano-4-hydroxycinnamic acid matrix in the mass range *m/z* 190.0499–749.5157 or 1046.5417. Accurately measured spectra were internally calibrated and elemental analysis was performed on Data Explorer v. 4.9 Software with mass accuracy better than 5 ppm. Samples were prepared by mixing 1 lμL of analyte methanol solution with 5 μL of saturated (10 mg/mL) solution of a-cyano 4-hydroxycinnamic acid (a-CHCA) and internal calibrants (0.1 mg/mL) dissolved in 50 % acetonitrile/0.1 % TFA. Microwave assisted reactions were performed on CEM Discovery System with infrared temperature control. The reaction mixtures were placed in a flask equipped with a magnetic stir bar and subjected to microwave irradiation.

*N*^α^-benzyl-*N*^β^-Boc amino acids **1** were prepared according to procedure described by Lelais and Seebach (2003). *N*-benzyl-α-amino acid (1 equiv.) was dissolved in dry MeOH and (CH_3_)_4_NOH (1 equiv.) was added at 0 °C. After 30 min solvent was evaporated and the residue dissolved in dry CH_2_Cl_2_. The solution was cooled to −78 °C and *N*-Boc-3-trichloromethyloxaziridine (1.3 equiv., prepared according to Vidal et al. (1998)) dissolved in CH_2_Cl_2_ was added dropwise. Reaction was stirred at room temperature overnight. Solution was washed three times with water, collected water layers were acidified with KHSO_4_ to pH 3 and product extracted with CH_2_Cl_2_. Solvent was evaporated and the product (yellow oil, 40 %) used without further purification. α-Hydrazino acids were prepared according the procedure described by Panda et al. (2013), with slight modifications. d-Amino acid was dissolved in 2.5 M H_2_SO_4_ (1.3 mL/mmol) and KBr (3.5 equiv.) was added. The solution was cooled down to 0 °C and then solution of NaNO_2_ (1.3 equiv.) was added dropwise. After 1 h at 0 °C, the reaction mixture was stirred at room temperature overnight. α-Bromo acid was extracted with EtOAc, washed with NaHCO_3_ and dried over MgSO_4_. After evaporation, product was obtained as yellow oil (85 % yield.). Obtained α-bromo acid was dissolved in EtOH (2 mL) and added dropwise to solution of hydrazine hydrate (3 equiv.) in EtOH (1 mL). The reaction mixture was irradiated under MW at 70 °C and 50 W for 1 h. A white suspension was obtained. Solvent was evaporated, and product recrystallized from EtOH/ether (40 % yield). Optical purity of prepared hydrazino acids has been checked by the chiral thin-layer chromatography and confirmed complete conversion.

### General procedure for the synthesis of dipeptides **2**

Dipeptides were prepared by acid mediated removal of the Boc group from the corresponding N-terminally protected dipeptides (TFA-water 9:1, v/v; 30 min.; r.t.; quant.). N-terminally protected dipeptides were obtained by the following procedure: Boc-Aaa-OH (Aaa = Leu, Val, Ala) (1 mmol) and HOSu (1.5 mmol) were dissolved in dry DMF (3 mL) and solution cooled down to 0 °C. DCC (1.5 mmol) dissolved in dry DMF (2 mL) was added dropwise. After 30 min reaction was stirred at room temperature and the consumption of starting dipeptide followed by TLC. The precipitate was filtered, and the filtrate added dropwise to the solution of H-Phe-R (R=OH, OEt or NH_2_) (1 mmol) and KHCO_3_ (2 mmol) in water (5 mL). Reaction mixture was stirred at room temperature overnight. Solvent was evaporated and the residue purified by the flash column chromatography. Mobile phase used for the chromatography was also used for the determination of *R*_f_ value and is given for each compound.

### Boc-Leu-Phe-OH

Colourless oil (0.68 g, 82 %); *R*_f_ 0.40 (petrol ether-EtOAc-AcOH 7:5:0.5). ^1^H NMR (300 MHz, [D_6_]DMSO, 25 °C): δ = 12.6 (br s, 1H, OH), 7.88 (d, ^3^*J*_NH,H_ = 7.9 Hz, 1H, NH Phe), 7.31–7.10 (m, 5H, δ, ε, ζ Phe), 6.83 (d, ^3^*J*_NH,H_ = 8.6 Hz, 1H, NH Leu), 4.49–4.38 (m, 1H, α Phe), 4.00–3.88 (m, 1H, α Leu), 3.05; 2.90 (dd, ^3^*J*_H,H_ = 5.1 Hz, ^3^*J*_H,H_ = 8.6 Hz, ^2^*J*_H,H_ = 13.9 Hz, 2H, β, β′ Phe), 1.55–1.46 (m, 3H, β, β′, γ Leu), 1.36 (s, 9H, CH_3_ Boc), 0.83; 0.80 (d, ^3^*J*_H,H_ = 6.6 Hz, 6H, δ, δ′ Leu). ^13^C NMR (300 MHz, [D_6_]DMSO, 25 °C): δ = 172.8 (CO Phe), 172.3 (CO Leu), 155.1 (CO Boc), 137.3 (γ Phe), 129.0 (δ Phe), 128.0 (ε Phe), 126.4 (ζ Phe), 78.0 (C Boc), 53.1 (α Phe), 52.8 (α Leu), 41.0 (β Leu), 36.7 (β Phe), 28.1 (CH_3_ Boc), 24.1 (γ Leu), 22.8; 21.6 (δ, δ′ Leu).

### Boc-Val-Phe-OH

Colourless oil (0.45 g, 54 %); *R*_f_ 0.38 (petrol ether-EtOAc-AcOH 7:5:0.5). ^1^H NMR (300 MHz, [D_6_]DMSO, 25 °C): δ = 8.04 (d, ^3^*J*_NH,H_ = 7.8 Hz, 1H, NH Phe), 7.29–7.10 (m, 5 H, δ, ε, ζ Phe), 6.57 (d, ^3^*J*_NH,H_ = 8.2 Hz, 1H, NH Val), 4.42 (m, 1H, α Phe), 3.76 (m, 1H, α Val), 3.05; 2.88 (dd, ^3^*J*_H,H_ = 5.2 Hz, ^3^*J*_H,H_ = 8.5 Hz, ^2^*J*_H,H_ = 13.8 Hz, 2H, β, β′ Phe), 1.84 (m, 1H, β Val), 1.37 (s, 9H, CH_3_ Boc), 0.74; 0.77 (d, ^3^*J*_H,H_ = 6.6 Hz, 6H, γ, γ′ Val). ^13^C NMR (300 MHz, [D_6_]DMSO, 25 °C): δ = 172.8 (CO Phe), 171.3 (CO Val), 155.2 (CO Boc), 137.0 (γ Phe), 129.1 (δ Phe), 128,1 (ε Phe), 126.4 (ζ Phe), 78.1 (C Boc), 59.7 (α Val), 53.2 (α Phe), 36.8 (β Phe), 30.5 (β Val), 28.2 (CH_3_ Boc), 19.1; 18.1 (γ, γ′ Val).

### Boc-Ala-Phe-OH

Colourless oil (1.78 g, 36 %); *R*_f_ 0.30 (petrol ether-EtOAc-AcOH 7:5:0.5). ^1^H NMR (300 MHz, [D_6_]DMSO, 25 °C): δ = 7.83 (d, ^3^*J*_NH,H_ = 7.7 Hz, 1H, NH Phe), 7.30–7.15 (m, 5H, δ, ε. ζ Phe), 6.81 (d, ^3^*J*_NH,H_ = 6.5 Hz, 1H, NH Ala), 4.40 (m, 1H, α Phe), 3.95 (m, 1H, α Ala), 3.04; 2.91 (dd, ^3^*J*_H,H_ = 5.1 Hz, ^3^*J*_H,H_ = 8.4 Hz, ^2^*J*_H,H_ = 13.9 Hz, 2H, β, β′ Phe), 1.36 (s, 9H, CH_3_ Boc), 1.12 (d, ^3^*J*_H,H_ = 7.0 Hz, 3H, β Ala). ^13^C NMR (300 MHz, [D_6_]DMSO, 25 °C): δ = 172.6 (CO Phe), 172.5 (CO Ala), 154.8 (CO Boc), 137.3 (γ Phe), 129.1 (δ Phe), 128.0 (ε Phe), 126.3 (ζ Phe), 77.5 (C Boc), 53.1 (α Phe), 49.6 (α Ala), 18.1 (β Ala), 36.7 (β Phe), 28.1 (CH_3_ Boc).

### Boc-Leu-Phe-NH_2_

White powder (0.31 g, 83 %); m.p. 140 °C; *R*_f_ 0.35 petrol ether:EtOAc:AcOH (10:5:0.5). ^1^H NMR (300 MHz, [D_6_]DMSO, 25 °C): δ = 7.70 (d, ^3^*J*_NH,H_ = 7.8 Hz, 1H, NH Phe), 7.31–7.14 (m, 5H, δ, ε, ξ Phe), 7.36; 7.12 (br s, 2H, CONH_2_), 6.96 (d, ^3^*J*_NH,H_ = 8.5 Hz, 1H, NH Leu), 4.45 (m, 1H, α Phe), 3.84 (m, 1H, α Leu), 3.00–2.82 (m, 2H, β, β′ Phe), 1.79–1.55 (m, 2H, β, β′ Leu), 1.55–1.44 (m, 1H, γ Leu), 1.37 (s, 9H, CH_3_ Boc), 0.84; 0.80 (d, ^3^*J*_H,H_ = 6.6 Hz, 6H δ, δ′ Leu). ^13^C NMR (300 MHz, [D_6_]DMSO, 25 °C): δ = 172.5 (CO Leu), 155.7 (CO Boc), 137.2 (γ Phe), 129.1 (δ Phe), 128.0 (ε Phe), 126.3 (ζ Phe), 78.6 (C Boc), 53.9 (α Phe), 53.6 (α Leu), 48.0 (β Leu), 38.1 (β Phe), 28.1 (CH_3_ Boc), 24.6 (γ Leu), 23.3; 22.1 (δ, δ′ Leu).

### Boc-Val-Phe-NH_2_

White powder (0.33 g, 90 %); m.p. 148 °C; *R*_f_ 0.33 (petrol ether:EtOAc:AcOH 10:5:0.5). ^1^H NMR (300 MHz, [D_6_]DMSO, 25 °C): δ = 7.75 (d, ^3^*J*_NH,H_ = 7.7 Hz, 1H, NH Phe), 7.30–7.14 (m, 5H, δ, ε, ξ Phe), 7.32; 7.02 (br s, 2H, CONH_2_), 6.64 (d, ^3^*J*_NH,H_ = 8.0 Hz, 1H, NH Val), 4.60–4.35 (m, 1H, α Phe), 3.75–3.60 (m, 1H, α Val), 2.96; 2.83 (dd, ^3^*J*_H,H_ = 5.0 Hz, ^3^*J*_H,H_ = 8.5 Hz, ^2^*J*_H,H_ = 13.8 Hz, 2H, β, β′ Phe), 1.75–1.68 (m, 1H, β Val), 1.37 (s, 9H, CH_3_ Boc), 0.72; 0.68 (d, ^3^*J*_H,H_ = 6.6 Hz, 6H, γ, γ′ Val). ^13^C NMR (300 MHz, [D_6_]DMSO, 25 °C): δ = 173.2 (CO Val), 156.3 (CO Boc), 137.0 (γ Phe), 129.2 (δ Phe), 128.0 (ε Phe), 126.2 (ζ Phe), 78.6 (C Boc), 60.2 (α Val), 54.0 (α Phe), 37.6 (β Phe), 30.7 (β Val), 28.6 (CH_3_ Boc), 19.1; 18.1 (γ, γ′ Val).

### Boc-Ala-Phe-NH_2_

White powder (0.24 g, 72 %); m.p. 157 °C; *R*_f_ 0.14 (petrol ether:EtOAc:AcOH 10:5:0.5). ^1^H NMR (300 MHz, [D_6_]DMSO, 25 °C): δ = 7.65 (d, ^3^*J*_NH,H_ = 7.8 Hz, 1H, NH Phe), 7.25–7.15 (m, 5H, δ, ε. ζ Phe), 7.00 (d, ^3^*J*_NH,H_ = 6.4 Hz, 1H, NH Ala), 7.45; 7.10 (br s, 2H, CONH_2_), 4.43 (m, 1H, α Phe), 3.87 (m, 1H, α Ala), 3.04–2.91 (m, 2H, β, β′ Phe), 1.36 (s, 9H, CH_3_ Boc), 1.07 (d, ^3^*J*_H,H_ = 6.8 Hz, 3H, β Ala). ^13^C NMR (300 MHz, [D_6_]DMSO, 25 °C): δ = 172.6 (CO Phe), 172.3 (CO Ala), 156.6 (CO Boc), 137.1 (γ Phe), 129.0 (δ Phe), 128.1 (ε Phe), 126.3 (ζ Phe), 78.1 (C Boc), 53.2 (α Phe), 47.3 (α Ala), 36.7 (β Phe), 28.1 (CH_3_ Boc), 18.0 (β Ala).

### General procedure for the synthesis of tripeptides **3a** and **3b**

*N*^α^-benzyl-*N*^β^-Boc amino acid **1** (0.5 mmol) was dissolved in dry DMF, NMM (0.5 mmol), BOP (0.55 mmol) and HOBt (0.55 mmol) were added. After 30 min solution of dipeptide **2** (0.5 mmol) and NMM (0.5 mmol) in dry DMF (1 mL) was added. Reaction was stirred at room temperature overnight. Solvent was evaporated and the residue purified by the flash column chromatography. Mobile phase used for the chromatography was also used for the determination of *R*_f_ value and is given for each compound.

### *N*^α^-benzyl-*N*^β^-Boc-Leu-Leu-Phe-OH (**3a**)

Yellow oil (56 mg, 19 %); *R*_f_ 0.66 (petrol ether:EtOAc:AcOH 5:5:0.5). ^1^H NMR (600 MHz, [D_6_]DMSO, 25 °C): δ = 8.29 (d, ^3^*J*_NH,H_ = 7.9 Hz, 1H, NH Phe), 7.88 (d, ^3^*J*_NH,H_ = 8.8 Hz, 1H, NH), 7.78 (br s, 1H, NH), 7.65–7.55 (m, 5H, Bn), 7.26–7.16 (m, 5H δ, ε, ξ Phe), 4.53 (m, 1H, α Phe), 4.43 (m, 1H, α Leu), 4.34 (m, 1H, α hLeu), 3.79 (s, 2H, CH_2_ Bn), 3.00–2.89 (m, 2H β, β′ Phe), 1.53 (m, 3H, β, γ Leu), 1.42–1.34 (m, 3H, β, γ hLeu), 1.33–1.25 (m, 9H CH_3_ Boc), 0.84–0.74 (m, 12H, δ, δ′, Leu, hLeu). ^13^C NMR (151 MHz, [D_6_]DMSO, 25 °C): δ = 172.9, 171.6, 170.4 (CO Phe, Leu, hLeu), 154.9 (CO Boc), 137.6 (C Bn), 137.4 (γ Phe), 133.1, 132.4, 131.4 (CH Bn), 129.1, 128.1, 127.0 (δ, ε, ζ Phe), 78.2 (α hLeu), 60.1 (α Phe), 53.2 (CH_2_ Bn), 50.7 (α Leu), 37.0 (β Leu), 36.8 (β Phe), 36.6 (β hLeu), 28.0 (CH_3_ Boc), 24.2 (γ hLeu), 23.8 (γ Leu), 23.0 (δ hLeu), 22.8 (δ Leu), 21.7 (δ′ hLeu), 21.4 (δ′ Leu). HRMS (MALDI-TOF/TOF): calcd. for C_33_H_48_N_4_O_6_ [M + Na]^+^ 619.3466; found 619.3446.

### *N*^α^-benzyl-*N*^β^-Boc-Val-Val-Phe-OH (**3b**)

Yellow oil (66 mg, 12 %); *R*_f_ 0.50 (petrol ether:EtOAc:AcOH 10:5:0.5). ^1^H NMR (600 MHz, [D_6_]DMSO, 25 °C): δ = 7.62–7.56 (m, 5H, Bn), 7.45–7.24 (m, 5H, δ, ε, ξ Phe), 4.43 (m, 1H, α Phe), 4.33–4.12 (m, 2H, α Val, hVal), 3.84–3.66 (m, 2H, CH_2_ Bn), 3.05–2.95 (m, 2H, β Phe), 2.02–1.85 (m, 2H, β Val, hVal), 1.29–1.17 (m, 9H, CH_3_ Boc), 0.93–0.70 (m, 12H, γ, γ′ Val, hVal). ^13^C NMR (151 MHz, [D_6_]DMSO, 25 °C): δ = 172.7, 170.6, 163.8 (CO Phe, Val, hVal), 154.3 (CO Boc), 137.5 (C Bn), 137.4 (γ Phe), 133.1, 132.4, 131.4 (CH, Bn), 128.7, 128.0, 127.8 (δ, ε, ζ Phe), 78.6 (C Boc), 78.1 (α hVal), 60.8 (α Phe), 57.3 (CH_2_ Bn), 53.1 (α Val), 36.6 (β Phe), 34.7 (β hVal), 30.4 (β Val), 28.0 (CH_3_ Boc), 19.3, 18.8 (γ, γ′ hVal), 18.3, 17.9 (γ, γ′ Val). HRMS (MALDI-TOF/TOF): calcd. for C_31_H_44_N_4_O_6_ [M + K]^+^ 607.2892; found 607.291.

### General Procedure for the synthesis of tripeptides **3c**–**3g**

*N*^α^-benzyl-*N*^β^-Boc amino acid **1** (0.5 mmol) and dipeptide **2** (0.5 mmol) were dissolved in dry DMF; NMM (1 mmol), BOP (0.55 mmol) and HOBt (0.55 mmol) were added. Reaction was stirred at room temperature overnight. Solvent was evaporated and the residue purified by the flash column chromatography. Mobile phase used for the chromatography was also used for the determination of *R*_f_ value and is given for each compound.

### *N*^α^-benzyl-*N*^β^-Boc-Leu-Leu-Phe-OEt (**3c**)

Yellow oil (32 mg, 10 %); *R*_f_ 0.75 (petrol ether:EtOAc:AcOH 10:5:0.5). ^1^H NMR (600 MHz, [D_6_]DMSO, 25 °C): δ = 8.57 (d, ^3^*J*_NH,H_ = 7.4 Hz, 1H, NH), 8.32 (d, ^3^*J*_NH,H_ = 7.2 Hz, 1H, NH), 7.79 (d, ^3^*J*_NH,H_ = 7.5 Hz, 1H, NH), 7.42–7.08 (m, 10H, Bn, δ, ε, ξ Phe), 4.50–4.33 (m, 3H, α Phe, CH_2_ OEt), 4.07–3.95 (m, 2H, CH_2_ Bn), 3.80 (m, 1H, α Leu), 3.48–3.32 (m, 1H, α hLeu), 3.07–2.90 (m, 2H, β, β′ Phe), 1.66–1.40 (m, 6H, β, β′, γ Leu, hLeu), 1.40–1.17 (m, 9H, CH_3_ Boc), 1.12–1.06 (m, 3H, CH_3_ OEt), 0.93–0.75 (m, 12H, δ, δ′ Leu, hLeu). ^13^C NMR (151 MHz, [D_6_]DMSO, 25 °C): δ = 171.8, 171.2, 170.6 (CO Phe, Leu, hLeu), 156.0 (CO Boc), 137.5 (C Bn), 137.1 (γ Phe), 129.0, 128.8, 128.1 (CH Bn), 127.8, 127.0, 126.5 (δ, ε, ζ Phe), 78.2 (α hLeu), 76.3 (C Boc), 60.4 (CH_2_ OEt), 60.1 (CH_2_ Bn), 53.7 (α Leu), 53.5 (α Phe), 51.4 (β Phe), 40.1 (β hLeu), 36.4 (β Leu), 28.0 (CH_3_ Boc), 24.2 (γ hLeu), 24.0 (γ Leu), 22.9; 22.8 δ, δ′ hLeu), 21.5; 21.2 (δ, δ′ Leu), 13.8 (CH_3_ OEt). HRMS (MALDI-TOF/TOF): calcd. for C_35_H_52_N_4_O_6_ [M + Na]^+^ 647.3779; found 647.3766.

### *N*^α^-benzyl-*N*^β^-Boc-Val-Val-Phe-OEt (**3d**)

Yellow oil (82 mg, 27 %); *R*_f_ 0.71 (petrol ether:EtOAc:AcOH 10:5:0.5). ^1^H NMR (600 MHz, [D_6_]DMSO, 25 °C): δ = 8.65 (d, ^3^*J*_NH,H_ = 7.2 Hz, 1H, NH), 8.27 (d, ^3^*J*_NH,H_ = 7.3 Hz, 1H, NH), 7.65 (d, ^3^*J*_NH,H_ = 7.4 Hz, 1H, NH),7.66–7.53 (m, 5H, Bn), 7.31–7.16 (m, 5H, δ, ε, ξ Phe), 4.45 (m, 2H, CH_2_ OEt), 4.31–4.24 (m, 1H, α Phe), 4.23–4.17 (m, 1H, α Val), 4.17–4.10 (m, 1H, α hVal), 4.01 (m, 2H, CH_2_ Bn), 3.01–2.94 (m, 2H, β, β′ Phe), 1.93 (m, 1H, β hVal), 1.72 (m, 1H, β Val), 1.65–1.25 (m, 9H, CH_3_ Boc), 1.16–1.00 (m, 6H, γ, γ′ Val), 0.94–0.86 (m, 3H, CH_3_ OEt), 0.85–0.75 (m, 6H, γ, γ′ hVal). ^13^C NMR (151 MHz, [D_6_]DMSO, 25 °C): δ = 171.2, 170.7, 160.7 (CO Phe, Val, hVal), 156.6 (CO Boc), 133.1 (Bn), 132.4 (γ Phe), 132.1, 131.5, 131.4 (CH Bn), 129.0, 128.7, 128.1 (δ, ε, ζ Phe), 60.4 (α hVal), 57.2 (α Val), 55.5 (α Phe), 53.5 (CH_2_ OEt), 47.5 (β Bn), 36.5 (β Phe), 33.3 (β hVal), 30.7 (β Val), 28.0 (CH_3_ Boc), 25.3, 24.4 (γ, γ′ hVal), 19.1, 17.9 (γ, γ′ Val), 13.9 (CH_3_ OEt). HRMS (MALDI-TOF/TOF): calcd. for C_33_H_48_N_4_O_6_ [M + Na]^+^ 619.3466; found 619.3478.

### *N*^α^-benzyl-*N*^β^-Boc-Leu-Leu-Phe-NH_2_ (**3e**)

Yellow oil (212 mg, 71 %); *R*_f_ 0.48 (petrol ether:EtOAc:AcOH 7:5:0.5). ^1^H NMR (600 MHz, CD_3_OD, 25 °C): δ = 7.42–7.04 (m, 12H, δ, ε, ξ Phe, Bn, CONH_2_), 4.65; 4.58 (m, 1H, α Phe), 4.40; 4.19 (m, 1H, α Leu), 3.46; 3.32 (m, 1H, α hLeu), 3.14–2.77 (m, 2H, β, β′ Phe), 1.87–1.45 (m, 6H, β, β′, γ Leu, hLeu), 1.29 (br s, 9H, Boc), 0.94–0.80 (m, 12H, δ, δ′ Leu, hLeu). ^13^C NMR (151 MHz, CD_3_OD, 25 °C): δ = 175.8, 174.4, 172.6 (CO Phe, Leu, hLeu), 158.7 (CO Boc), 137.9 (γ Phe), 137.0, 129.6, 129.5, 129.4 (Bn), 129.1 (ε Phe), 128.5 (δ Phe), 127.8 (ε Phe), 80.8 (C Boc), 61.8 (CH_2_ Bn), 55.6, 55.5 (α Phe), 53.8, 53.0 (α Leu), 49.7 (α hLeu), 41.4, 41.1 (β Leu), 40.2, 40.1 (β Phe), 38.9, 38.3 (β hLeu), 28.6 (CH_3_ Boc), 25.8, 25.4 (γ Leu, hLeu), 23.4, 23.0, 22.5, 21. 8 (δ, δ′ Leu, hLeu). HRMS (MALDI-TOF/TOF): calcd. for C_33_H_49_N_5_O_5_ [M + Na]^+^ 618.3625; found 618.3618.

### *N*^α^-benzyl-*N*^β^-Boc-Val-Val-Phe-NH_2_ (**3f**)

Yellow oil (195 mg, 69 %); *R*_f_ 0.46 (petrol ether:EtOAc:AcOH 5:5:0.5). ^1^H NMR (600 MHz, [D_6_]DMSO, 25 °C): δ = 8.11, 7.96, 7.75 (br d, 3H, NH Phe, Val, hVal), 7.42-7.19 (m, 10H, Bn, Phe), 7.16 (br s, 2H, NH_2_), 4.49 (m, 1H, α Phe), 4.22 (m, 1H, α Val), 3.76 (m, 2H, CH_2_ Bn,), 3.08-2.91 (m, 2H, β, β′ Phe), 2.81 (m, 1H, α hVal), 1.96 (m, 1H, β Val), 1.86 (m, 1H, β hVal), 1.32-1.13 (br s, 9H CH_3_ Boc), 1.07-0.65 (m, 12H, γ, γ′ Val, hVal). ^13^C NMR (151 MHz, [D_6_]DMSO, 25 °C): δ = 172.6 (CO Phe), 170.3, 170.1 (CO Val, hVal), 163.7 (CO Boc), 137.7 (γ Phe),), 137.6 (C Bn), 132.0, 131.5, 128.7 (CH Bn), 128.0, 127.8, 126.9, (δ, ε, ζ Phe), 78.1 (C Boc), 60.3 (CH_2_ Bn), 57.6 (α Val), 53.5 (α Phe), 37.6 (β Phe), 30.2 (β Val), 28.0 (β hVal), 19.3 (CH_3_ Boc), 18.9, 18.3 (γ, γ′ Val, hVal). HRMS (MALDI-TOF/TOF): calcd. for C_31_H_45_N_5_O_5_ [M + Na]^+^ 590.3313; found 590.3334.

### *N*^α^-benzyl-*N*^β^-Boc-Ala-Ala-Phe-NH_2_ (**3g**)

Yellow oil: (176 mg, 69 %); *R*_f_ 0.31 (EtOAc:EtOH:AcOH:H_2_O 70:10:2:2). ^1^H NMR (600 MHz, [D_6_]DMSO, 25 °C): δ = 7.40–7.35 (br s, 3H. NH Ala, hAla, Phe), 7.30–7.18 (m, 10H, Bn, Phe), 7.15 (s, 2H, NH_2_), 4.41 (m, 1H, α Phe), 4.22 (m, 1H, α Ala), 3.82 (s, 2H, CH_2_ Bn), 3.57 (m, 1H, α hAla), 3.04–2.81 (m, 2 H, β, β′ Phe), 1.40 (m, 6H, β Ala, hAla), 1.22 (s, 9H, CH_3_ Boc). ^13^C NMR (151 MHz, [D_6_]DMSO, 25 °C): δ = 172.6 (CO Phe), 171.9 (CO, Ala), 171.6 (CO, hAla), 137.8 (Bn), 136.9 (γ Phe), 129.2, 128.2, 127.9 (CH Bn), 127.8, 127.0, 126.8, (δ, ε, ζ Phe), 78.5 (C Boc), 62.2 (α hAla), 53.6 (α Phe), 48.0 (α Ala), 37.4 (β Phe), 28.2 (CH_3_ Boc), 21.0 (β Ala), 17.8 (β hAla). HRMS (MALDI-TOF/TOF): calcd. for C_27_H_37_N_5_O_5_ [M + Na]^+^ 534.2686; found 534.2699.

### General procedure for the synthesis of *N*^α^-benzyl-*N*^β^-Boc-Aaa-Gly-OH **4a** and **4b**

*N*^α^-benzyl-*N*^β^-Boc-Aaa-OH (Aaa = Leu, Val) (0.45 mmol) and HOSu (0.5 mmol) were dissolved in 3 mL dry DMF and solution cooled down to 0 °C. DCC (0.5 mmol) dissolved in 2 mL dry DMF was added dropwise. After 30 min reaction was stirred at room temperature and the consumption of starting dipeptide followed by TLC. The precipitate was filtered, and the filtrate added dropwise to the solution of glycine (0.45 mmol) and KHCO_3_ (0.45 mmol) in 5 mL of water. Reaction mixture was stirred at room temperature overnight. Solvent was evaporated and the residue purified by the flash column chromatography. Mobile phase used for the chromatography was also used for the determination of *R*_f_ value and is given for each compound.

### *N*^α^-benzyl-*N*^β^-Boc-Leu-Gly-OH (**4a**)

Yellow oil: (117 mg, 52 %); *R*_f_ 0.57 (EtOAc:petrol ether:AcOH 10:5:0.5). ^1^H NMR (600 MHz, CDCl_3_, 25 °C): δ = 7.57–7.03 (m, 5H, Bn), 5.52 (s, 2H, CH_2_ Bn), 4.12 (br s, 2H, α Gly), 3.46–3.36 (m, 1H, α Leu), 1.95–1.88 (m, 2H, β Leu), 1.59 (m, 1H, γ Leu), 1.54–1.21 (m, 9H, CH_3_ Boc), 1.15–0.75 (m, 6H, δ, δ′ Leu). ^13^C NMR (151 MHz, CDCl_3_, 25 °C): δ = 175.8 (CO Gly), 163.1 (CO Leu), 157.9 (CO Boc), 129.7, 128.9, 128.0 (CH Bn), 64.0 (α Leu), 61.3 (CH_2_ Bn), 49.7 (α Gly), 36.9 (β Leu), 28.5 (CH_3_ Boc), 25.2 (γ Leu), 21.1, 21.0 (δ, δ′ Leu). HRMS (MALDI-TOF/TOF): calcd. for C_20_H_31_N_3_O_5_ [M + Na]^+^ 416.2156; found 416.2166.

### *N*^α^-benzyl-*N*^β^-Boc-Val-Gly-OH (**4b**)

Yellow oil (82 mg, 46 %); *R*_f_ 0.70 (petrol ether:EtOAc:AcOH 7:5:0.5). ^1^H NMR (600 MHz, [D_6_]DMSO, 25 °C): δ = 8.51–7.09 (m, 5H, Bn), 5.09 (s, 2H, CH_2_ Bn), 3.96–3.68 (m, 1H, α Val), 3.60–2.96 (m, 2H α Gly), 1.39 (m, 1H, β Val), 1.32–1.18 (m, 9H, CH_3_ Boc), 1.08–0.99 (m, 6H, γ, γ′ Val). ^13^C NMR (151 MHz, [D_6_]DMSO, 25 °C): δ = 173.5 (CO Gly), 171.0 (CO Val), 156.6 (CO Boc), 128.9, 127.8, 126.8 (CH Bn), 78.6 (C Boc), 76.8 (α Val), 65.5 (CH_2_ Bn), 47.5 (α Gly), 28.0 (CH_3_ Boc), 25.0 (β Val), 19.3, 18.2 (γ, γ′ Val). HRMS (MALDI-TOF/TOF): calcd. for C_19_H_29_N_3_O_5_ [M + Na]^+^ 402.1999; found 402.2005.

### Synthesis of *N*^α^-benzyl-*N*^β^-Boc-Leu-OMe (**5a**)

*N*^α^-benzyl-*N*^β^-Boc-Leu-OH (100 mg, 0.3 mmol) was dissolved in dry DMF (10 mL), KHCO_3_ (60 mg, 0.59 mmol) was added and then CH_3_I (30 μL, 0.48 mmol) dropwise. Reaction mixture was stirred at room temperature overnight. Water was added to the reaction and product extracted with EtOAc. Yellow oil: (102 mg, 95 %); *R*_f_ 0.64 (petrol ether:EtOAc:AcOH 15:5:0.5). ESI–MS: *m/z* 373 [M + Na]^+^.

### Synthesis of *N*^α^-benzyl-*N*^β^-Boc-Ala-OMe (**5b**)

*N*^α^-benzyl-*N*^β^-Boc-Ala-OH (100 mg, 0.3 mmol) was dissolved in dry DMF (10 mL), KHCO_3_ (60 mg, 0.59 mmol) was added and then CH_3_I (30 μL, 0.48 mmol) dropwise. Reaction mixture was stirred at room temperature overnight. Water was added to the reaction and product extracted with EtOAc. Yellow oil (109 mg, 100 %); *R*_f_ 0.83 (petrol ether:EtOAc:AcOH 7:5:0.5). ^1^H NMR (600 MHz, CD_2_Cl_2_, 25 °C): δ = 7.32 (br d, 1H, NH hAla), 7.40–7.24 (m, 5H, Bn), 4.02–3.91 (m, 2H, CH_2_ Bn), 3.71 (s, 3H, OCH_3_), 3.63 (m, 1H, α hAla), 1.36 (d, ^3^*J*_H,H_ = 7.3 Hz, 3H, β hAla), 1.34 (s, 9H, CH_3_ Boc). ^13^C NMR (151 MHz, CD_2_Cl_2_, 25 °C): δ = 164.6 (CO Ala), 137.8 (C Bn), 132.5, 128.7, 127.9 (CH Bn), 79.8 (C Boc), 76.5 (α hAla), 61.8 (CH_2_ Bn), 52.1 (OCH_3_), 28.5 (CH_3_ Boc), 16.6 (β hAla). ESI–MS: *m/z* 331 [M + Na]^+^, *m/z* 209 [M-Boc]^+^.

### Synthesis of *N*^α^-benzyl-hLeu-OMe (**6a**)

Crude product **5a** was dissolved in TFA-water 9:1, v/v) and the reaction was stirred at room temperature 60 min. Solvent was evaporated and the residue dried in vacuum.

### Synthesis of *N*^α^-benzyl-*N*^β^-Boc-Leu-*N*^α^-benzyl-Leu-OMe (**7**)

*N*^α^-benzyl-*N*^β^-Boc-Leu-OH (100 mg; 0.3 mmol) was dissolved in dry DMF, NMM (55 μL, 0.50 mmol) and HATU (125 mg, 0.33 mmol) were added. After 15 min solution of *N*^α^-benzyl-hydrazino leucine (100 mg; 0.3 mmol) and NMM (55 μL, 0.50 mmol) in 1 mL dry DMF was added. Reaction was stirred at room temperature overnight. Solvent was evaporated and the residue purified by flash column chromatography in petrol ether:EtOAc:AcOH 10:5:0.5. Yellow oil: (80 mg, 47 %); *R*_f_ 0.53 (petrol ether:EtOAc:AcOH 15:5:0.5). ESI–MS: [M + H]^+^*m/z* 569.3; [M + Na]^+^*m/z* 591.3. ^1^H NMR (600 MHz, CD_2_Cl_2_, 25 °C): δ = 7.49–7.14 (m, 10H, Bn), 6.86 (br s, 1H, NH), 5.83 (d, ^3^*J*_NH,H_ = 10.5 Hz, 1H, NH), 3.99–3.94 (m, 2H, α hLeu), 3.78–3.62 (m, 7 H, CH_2_, Bn, CH_3_ O–CH_3_), 1.63–1.56 (m, 4H, β, β′ hLeu), 1.38–1.31 (m, 9H, CH_3_ Boc), 0.99–0.87 (m, 14H, γ, δ, δ′ hLeu). ^13^C NMR (151 MHz, CD_2_Cl_2_, 25 °C): δ = 172.8, 172.7 (CO hLeu), 137.1, 137.0 (C Bn), 128.9, 128.1, 127.7, 127.6, 127.0, 126.0 (CH Bn), 80.9 (C Boc), 66.0 (CH_2_ Bn), 66.0, 62.2 (α hLeu), 60.6 (CH_2_ Bn), 50.9 (OCH_3_), 38.7, 38.5 (β hLeu), 27.4 (CH_3_ Boc), 24.2, 23.7 (γ Leu), 22.1, 20.9 (δ, δ′, hLeu). HRMS (MALDI-TOF/TOF): calcd. for C_32_H_48_N4_6_O_5_ [M + K]^+^ 607.3256; found 607.3256.

### Synthesis of *N*^β^-Boc-Leu-OMe (**8a**)

*N*^α^-benzyl-*N*^β^-Boc-Leu-OMe (240 mg, 0.71 mmol) was dissolved dry MeOH (45 mL), acetic acid (3 mL) and 10 % Pd/C (90 mg) were added. Reaction was performed under 15 atm H_2_ at room temperature for 3 days. Catalyst was filtered off, solvent evaporated and the residue purified by flash chromatography. Yellow oil: (88 mg; 48 %); *R*_f_ = 0.58 (petrol ether:EtOAc:AcOH 15:5:0.5). ESI–MS: [M-Boc]^+^*m/z* 161.2; [M + H]^+^*m/z* 261.2.

### Synthesis of *N*^β^-Boc-Phe-OMe (**8b**)

*N*^α^-benzyl-*N*^β^-Boc-Phe-OMe (100 mg, 0.26 mmol) was dissolved in dry MeOH (40 mL), acetic acid (3 mL) and 10 % Pd/C (26 mg) were added Reaction was performed under 15 atm H_2_ at room temperature for 3 days. Catalyst was filtered off, solvent evaporated and the residue purified by the flash column chromatography. Yellow oil: (57 mg; 74 %); *R*_f_ = 0.44 (petrol ether:EtOAc:AcOH 15:5:0.5). ^1^H NMR (600 MHz, CD_2_Cl_2_, 25 °C): δ = 7.33–7.22 (m, 6H, NH, δ, ε, ζ Phe), 6.16 (br. s, 1H, NH Phe), 3.93 (m, 1H, α hPhe), 3.69 (s, 3H, O–CH_3_), 3.05, 2.91 (m, 2H, β, β′ hPhe), 1.40 (s, 9H, CH_3_ Boc). ^13^C NMR (151 MHz, CD_2_Cl_2_, 25 °C): δ = 172.4 (CO hPhe), 155.6 (CO Boc), 136.5 (γ hPhe), 128.7, 127.0, 126.3 (δ, ε, ζ hPhe), 79.8 (C Boc), 63.6 (α hPhe), 51.3 (OCH_3_), 36.4 (β hPhe), 27.4 (CH_3_ Boc). ESI–MS: [M-Boc]^+^*m/z* 195.1; [M + H]^+^*m/z* 295.1; [M + Na]^+^*m/z* 317.1; [2 M + H]^+^*m/z* 589.3; [2 M + Na]^+^*m/z* 611.3

### Synthesis of Boc-Lys(Boc)-hLeu-OH (**9**)

Boc-Lys(Boc)-OH (236 mg, 0.68 mmol) and HOSu (117 mg, 1.02 mmol) were dissolved in dry DMF (3 mL) and solution cooled down to 0 °C. DCC (210 mg, 1.02 mmol) dissolved in dry DMF (2 mL) was added dropwise. After 30 min reaction was stirred at room temperature and the consumption of starting dipeptide followed by TLC. The precipitate was filtered, and the filtrate added dropwise to the solution of hLeu (100 mg, 0.68 mmol) and KHCO_3_ (136 mg, 1.36 mmol) in water (5 mL). Reaction mixture was stirred at room temperature overnight. Solvent was evaporated and the residue purified by the flash column chromatography (mobile phase: EtOAc:AcOH 70:2). Yellow oil: (156 mg, 48 %); *R*_f_ 0.38 (EtOAc:AcOH 70:2). ESI–MS: [M + H]^+^*m/z* 475, [M-Boc]^+^*m/z* 375, [M-2Boc]^+^*m/z* 275. ^1^H NMR (600 MHz, CDCl_3_, 25 °C): δ = 4.83–4.62 (m, 1H, α Lys), 3.83–3.60 (m, 1H, α hLeu), 3.16–3.07 (m, 2H, ε Lys), 1.90–1.50 (m, 8H, β, δ Lys, β hLeu, γhLeu,), 1.42 (m, 18H, CH_3_ Boc), 1.33 (m, 2H, γ Lys), 0.99, 0.96 (d, ^3^*J*_H,H_ = 6.6 Hz, 6H, δ, δ′ hLeu). ^13^C NMR (151 MHz, CDCl_3_, 25 °C): δ = 175.4 (CO hLeu), 52.9 (α Lys), 47.7 (α hLeu), 40.3 (β hLeu), 39.6 (ε Lys), 32.3 (β Lys), 29.8 (δ Lys), 28.4 (CH_3_ Boc), 25.1 (γ hLeu), 23.1 (γ Lys), 22.7, 22.1 (δ, δ′ hLeu). HRMS (MALDI-TOF/TOF): calcd. for C_22_H_42_N_4_O_7_ [M + Na]^+^ 497.2945; found 497.2937.

### Synthesis of Boc-Lys(Boc)-hLeu-Leu-OH (**10**)

Compound **9** (100 mg, 0.21 mmol) and HOSu (37 mg, 0.32 mmol) were dissolved in dry DMF (3 mL) and solution cooled down to 0 °C. DCC (65 mg, 0.32 mmol) dissolved in dry DMF (2 mL) was added dropwise. After 30 min reaction was stirred at room temperature and the consumption of starting dipeptide followed by TLC. The precipitate was filtered, and the filtrate added dropwise to the solution of leucine (28 mg, 0.21 mmol) and KHCO_3_ (42 mg, 0.42 mmol) in water (5 mL). Reaction mixture was stirred at room temperature overnight. Solvent was evaporated and the residue purified by the flash column chromatography (mobile phase: EtOAc:AcOH 70:2). Yellow oil: (61 mg, 49 %); *R*_f_ 0.73 (EtOAc:AcOH 70:2). ESI–MS: [M + H]^+^*m/z* 588.7, [2 M + H]^+^*m/z* 1176.1. ^1^H NMR (600 MHz, CDCl_3_, 25 °C): δ = 5.27 (m, 1H, α Leu), 3.88–3.59 (m, 1H, α Lys), 3.46 (m, 1H, α hLeu), 3.12 (m, 2H, ε Lys), 1.75–1.68 (m, 2H, β Lys), 1.67–1.58 (m, 2H, δ Lys), 1.58–1.51 (m, 2H, β Leu, hLeu), 1.51–1.41 (m, 18H, CH_3_ Boc), 1.38–1.32 (m, 2H, γ Lys), 1.22–0.82 (m, 12H, δ, δ′ Leu, hLeu). ^13^C NMR (151 MHz, CDCl_3_, 25 °C): δ = 176.2 (CO Leu), 175.3 (CO hLeu), 171.7 (CO Lys), 157.2 (CO Boc), 76.5 (C Boc), 63.9 (α hLeu), 61.5 (α Lys), 55.5 (α Leu), 41.1 (ε Lys), 40.4 (β Leu), 33.8 (β hLeu), 31.5 (β Lys), 29.7 (δ Lys), 28.4, 28.3 (CH_3_ Boc), 25.6 (γ hLeu), 24.9 (γ Leu), 23.5 (γ Lys), 23.1, 23.0 (δ, δ′ hLeu), 22.7, 22.5 (δ, δ′ Leu). HRMS (MALDI-TOF/TOF): calcd. for C_28_H_53_N_5_O_8_ [M + K]^+^ 626.3524; found 626.3542.

### Synthesis of Boc-Lys(Boc)-hLeu-hLeu-OH **(11)**

Compound **9** (100 mg, 0.21 mmol) was dissolved in dry DMF; NMM (23 μL, 0.21 mmol) and HATU (88 mg, 0.23 mmol) were added. After 15 min solution of hLeu (31 mg, 0.21 mmol) and NMM (23 μL, 0.21 mmol) in dry DMF (1 mL) was added. Reaction was stirred at room temperature overnight. Solvent was evaporated and the residue purified by the flash column chromatography (mobile phase: EtOAc:AcOH 70:2). Yellow oil: (68 mg, 53 %); *R*_f_ 0.35 (EtOAc:AcOH 70:2). ESI–MS: [M + H]^+^*m/z* 603.6, [M-Boc]^+^*m/z* 503.6. ^1^H NMR (600 MHz, MeOD, 25 °C): δ = 3.96 (m, 1H, α Lys), 3.49 (m, 2H, α hLeu), 3.06 (m, 2H, ε Lys), 1.89 (m, 2H, β Lys), 1.77–1.73 (m, 2H, δ Lys), 1.66–1.55 (m, 6H, β, β′, γ hLeu), 1.47 (m, 18H, CH_3_ Boc), 1.41–1.37 (m, 2H, γ Lys,), 1.02–0.98 (m, 12H, δ, δ′, hLeu). ^13^C NMR (151 MHz, CDCl_3_, 25 °C): δ = 178.1 (CO hLeu), 172.1 (CO hLeu), 170.9 (CO Lys), 157.2 (CO Boc), 82.1 (C Boc), 79.1 (C Boc), 66.3 (α hLeu), 63.9 (α Lys), 57.8 (α hLeu), 41.3 (β hLeu), 40.1 (ε Lys), 38.6 (α hLeu), 33.8 (β Lys), 29.6 (δ Lys), 28.4, 28.3 (CH_3_ Boc), 25.6 (γ hLeu), 24.8 (γ hLeu), 22.8 (γ Lys), 22.1, 22.0, 21.9 (δ,δ′ hLeu). HRMS (MALDI-TOF/TOF): calcd. for C_28_H_54_N_6_O_8_ [M + Na]^+^ 625.3895; found 625.3914.

## Results and discussion

Hydrazino derivatives of leucine, valine and alanine were prepared by two procedures: (1) electrophilic amination of the corresponding *N*-benzyl-l-amino acid with *N*-Boc oxaziridine (Lelais and Seebach 2003), and (2) nucleophilic substitution of d-amino acid-derived α-bromo acid with hydrazine hydrate (Panda et al. 2013). While first procedure yielded *N*^α^-benzyl-*N*^β^-Boc amino acid derivatives in ≈40 % yield, second approach gave rise to unprotected hydrazino acids also in 40 % yield.

**Scheme 2:**
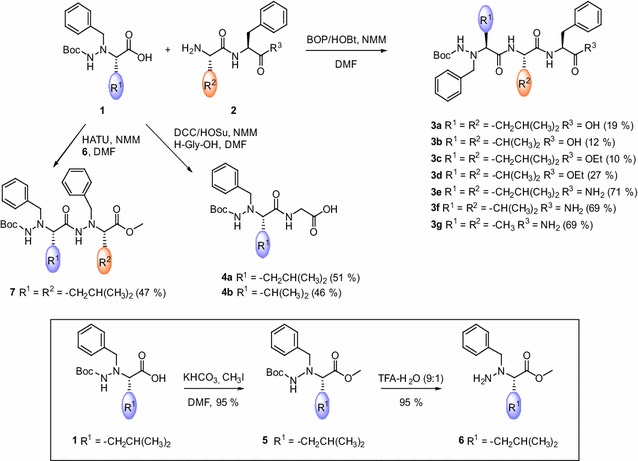
Synthesis of peptides based on *N* ^α^-benzyl hydrazino derivatives

The utility of *N*^α^-benzyl-*N*^β^-Boc amino acid derivatives as building blocks in synthesis of peptidomimetics was tested by coupling with Aaa-Phe dipeptides (Aaa = Leu, Val, Ala), carrying acid, ester or amide group at the C-terminus (Scheme 2). Guy et al. (1998) performed various activation of *N*^α^-benzyl-*N*^β^-Boc-Ala-OH and found PyBOP to be the most effective. Similarly, Lelais and Seebach (2003) observed that HATU activation is more effective that EDC/HOBt. We have tested activation of *N*^α^-benzyl-*N*^β^-Boc-l-leucine **1a** by mixed anhydride, DCC/HOSu, BOP and HATU, and only BOP and HATU activation gave products. However, free tripeptide acids were obtained in low yields (19 % for **3a** and 12 % for **3b**, Table 1). We then repeated couplings with ethyl ester of dipeptides **2** and gained corresponding tripeptide esters, again in low yields, (10 % for **3c** and 27 % for **3d**; Table 1). Switching to dipeptide amides turned out to be crucial for improving couplings; amides **3e**–**g** were obtained in ≈70 % yields (Table 1). Since the same carboxyl component, and the same coupling conditions were used in all examples, the reason for the observed difference was sought in conformation preferences of dipeptides **2.**

**Table 1:** Preparation of hydrazino tripeptides **3a**–**g**

Product	R^1^	R^2^	R^3^	Yield^a^
**3a**	–CH_2_CH(CH_3_)_2_	–CH_2_CH(CH_3_)_2_	OH	19
**3b**	–CH(CH_3_)_2_	–CH(CH_3_)_2_	OH	12
**3c**	–CH_2_CH(CH_3_)_2_	–CH_2_CH(CH_3_)_2_	OEt	10
**3d**	–CH(CH_3_)_2_	–CH(CH_3_)_2_	OEt	27
**3e**	–CH_2_CH(CH_3_)_2_	–CH_2_CH(CH_3_)_2_	NH_2_	71
**3f**	–CH(CH_3_)_2_	–CH(CH_3_)_2_	NH_2_	69
**3g**	–CH_3_	–CH_3_	NH_2_	69

^a^Isolated yields

**Fig. 1:**
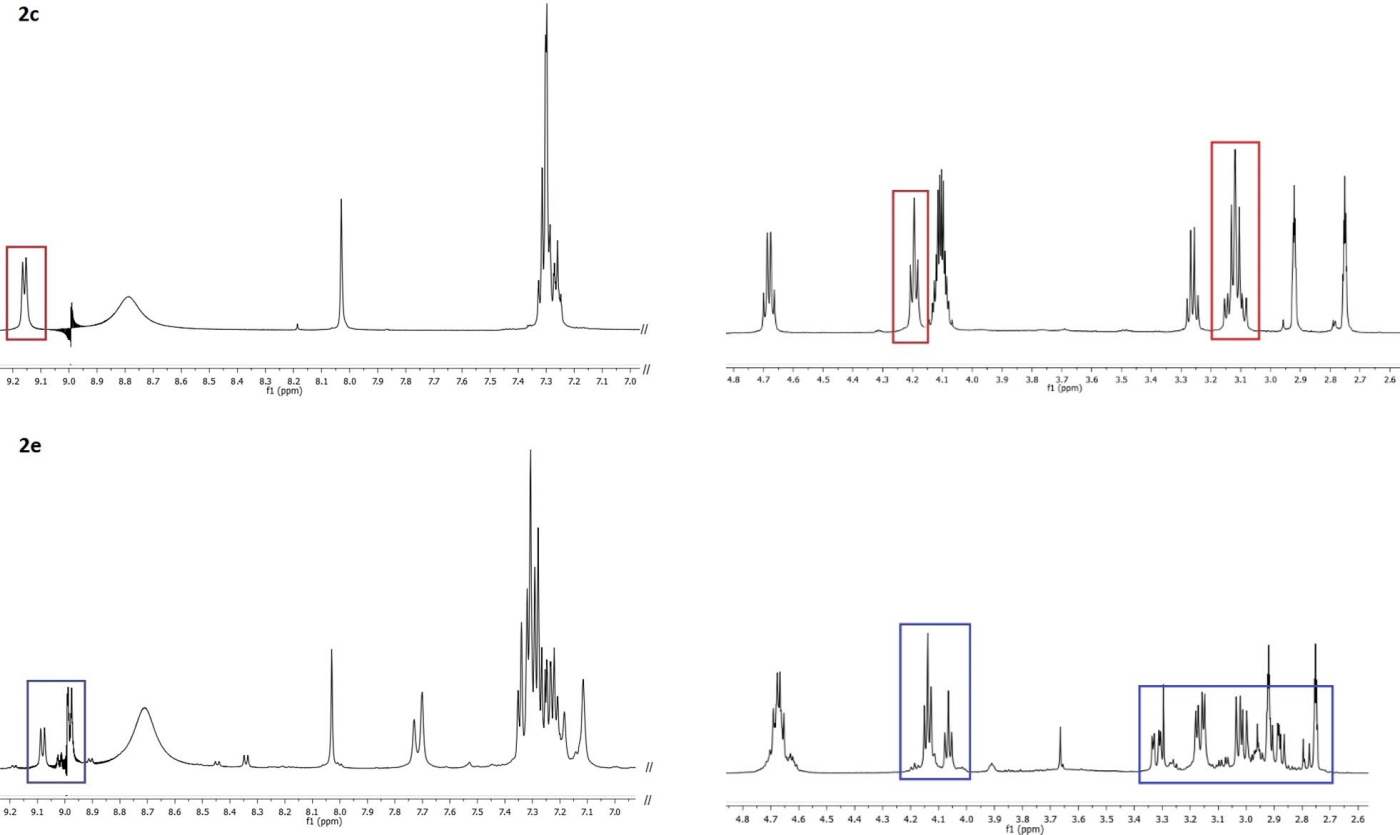
Parts of the ^1^H NMR spectra of dipeptide ester **2c** and dipeptide amide **2e **([D_7_]DMF) showing single or two sets of signals, respectively

Vijayadas et al. (2013) undertook crystallographic and NMR spectroscopic studies on a simple two-residue reversed-turn mimetics with C-terminal ester and amide groups and revealed that amides tend to form intramolecular hydrogen bond and stabilize folded conformation with higher propensity then the corresponding esters. However, these studies were performed in non-polar aprotic CDCl_3_, where intramolecular hydrogen bonds are expected. Close inspection of NMR spectra corresponding to dipepetide esters vs amides, revealed two set of signals in both ^1^H and ^13^C NMR spectra of dipeptide amides **2e**–**g**, while only one set of resonances is present in spectra of dipeptide esters **2c** and **2d**. Respective example is given at Fig. 1 for the ^1^H NMR spectra of ester **2c** and amide **2e.** Spectra were recorded in [D_7_]DMF to correspond closely to the conditions present during the peptide coupling. Two set of signals exhibit NH, Hα and Hβ, β′ protons of the Phe residue (Fig. 1), but also Leu side-chain protons (Additional file 1: Figure S1). Two set of signals, present in relative ratio 75:25, were assigned to *trans* and *cis* isomers of the Leu-Phe amide bond. ^1^H NMR spectrum of **2e** was also acquired at elevated temperatures (40, 60 and 80 °C; Additional file 1: Figure S2) and ratio of two sets of signals remains the same.

It is known that the free energy barrier for the *trans*–*cis* isomerization of peptidyl-prolyl amide bonds (Aaa-Pro) is in a range 60–100 kJ/mol, leading to the 5–40 % of *cis* isomer present in an unordered peptide chains (Jabs et al. 1999). Nonprolyl peptide bonds were found experimentally to contain about 0.5 % of *cis* isomer in dipeptides and about 0.15 % in longer peptides. Among them, aromatic amino acids are frequently found as residues flanking *cis* peptide bonds (Wawra and Fisher 2006). NMR study of Scherer et al. (1998) revealed that peptide bonds adjacent to the aromatic amino acid generate *cis* isomer population in a range 0.1–1 %, depending on a peptide length. Since NMR spectra of free dipeptide acids and dipeptide esters confirmed presence of a single conformer and those of dipeptide amides revealed presence of two conformers, it can be assumed that C-terminal amide group stabilizes *cis* conformation of the Aaa-Phe peptide bond. This stabilization is significant; having in mind that ratio of two isomers corresponds to those expected for the peptidyl–prolyl amide bonds (Wawra and Fisher 2006). It can be assumed that in polar solvents, like DMF, nucleophilic attack by the amino group on the activated carboxyl group of **1**, is more efficient in *cis* population of amides **2e**–**f**, since tripeptide amides are obtained in considerably higher yields than the corresponding esters of acids where only *trans* population is present. However, steric effects cannot be neglected. As a matter of fact, coupling of **1a** and **1b** with unprotected glycine gave corresponding hydrazino dipeptides **4a** and **4b** in fair yields (51 and 46 %, respectively; Scheme 2). Further elongation of peptide chain through coupling with *N*^α^-Bn-hLeu-OH gave only mixture of inseparable product, therefore, we decided to use C-terminal protected derivative, *N*^α^-Bn-hLeu-OMe and couple it with **1a**. Activation with HATU was performed and dipeptide **7** was isolated in 47 % yield.

**Scheme 3:**
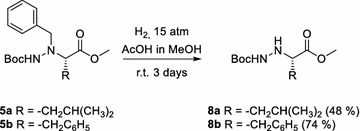
Deprotection of *N* ^α^-benzyl group in *N* ^α^-benzyl-*N* ^β^-Boc amino acids

Next, we examined deprotection of *N*^α^-benzyl-hydrazino units. It is known that catalytic hydrogenolysis of *N*-benzyl group proceeds slow and is often incomplete, or even unsuccessful. Interestingly, there are examples where Boc-deprotection or/and saponification of debenzylated Boc-protected peptide esters were ineffective (Lelais and Seebach 2003). We tested benzyl group removal on *N*^α^-benzyl-*N*^β^-Boc phenylalanine and leucine methyl esters and applied two procedures. Reaction with ammonium-formate in acidic media performed in reflux of methanol gave, after 3 days 32 % of debenzylated product, while 74 % of the desired product was obtained by catalytic hydrogenolysis. Reaction was performed under 15 atm H_2_ for 3 days at room temperature (Scheme 3).

Obtained results pointed to numerous obstacles associated with the usage of *N*^α^-benzyl hydrazino acids, starting from their incorporation into peptide chain to deprotection of the benzyl group. There are examples where coupling of *N*^α^-protected hydrazino derivatives to the activated amino acids, during solution and solid-phase synthesis was less effective (Bouillon et al. 2007a, b). Here we encountered problems with coupling to the activated *N*^α^-protected hydrazino acids and found that the conformational preferences of the nucleophile highly influence the outcome of the coupling reaction.

**Scheme 4:**
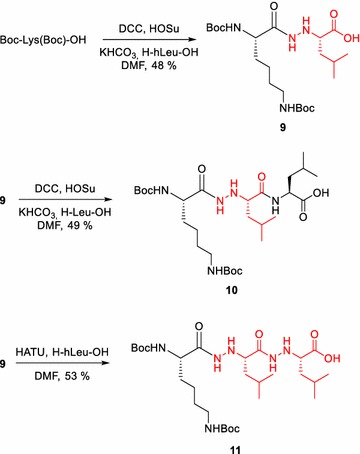
Synthesis of peptides based on hydrazino derivative of l-leucine

Therefore, we turned out attention to hydrazino acids (hAaa) obtained by the second approach. Panda et al. (2013) have recently shown that benzotriazolidines of the *N*^β^-Cbz hydrazino acids undergo acylation with chiral *N*-, O-, S, and C-nucleophiles. We further explored the utility of hydrazino derivatives of α-amino acids through series of couplings presented at Scheme 4. First we tested coupling of Boc-Lys(Boc)-OH and C-terminally unprotected hydrazino derivative of l-leucine (hLeu) and obtained product **9** in 48 % yield. Encouraged by this result, we performed two additional reactions to elongate peptide chain. Coupling of hydrazino dipeptide **9** with Leu through DCC/HOSu activation gave hydrazino tripeptide **10** in 49 % yield, while coupling with hLeu in the presence of HATU gave hydrazino tripeptide **11** in 53 % yield. It is know that activation of unprotected hydrazino acids may encounter side reactions leading to undesired diketopiperazines or various oligomers (Bently and Morley 1966; Guy et al. 1998; Maraud and Vanderesse 2004). Guy et al. (1998) found that activation with DCC/HOSu can be successful only if activated ester is formed in situ. Also, acylation with unprotected hydrazino acids took place regioselectively on the *N*^β^ when both amino acid partners bear bulky side chains. Similarly as Panda et al. (2013), and Acherer et al. (2013) we did not observe indication of oligomerization products under the applied reaction condition. The presence of bulky side chains and relatively efficient activation of the carboxyl component most probably contributed to this result. Acherar et al. (2013) have previously prepared mixed [α/α-hydrazino]tetra- and octamer using C-terminally protected hAla-OMe. Here we showed that acylation reactions proceed smoothly with fully unprotected nucleophiles, thus allowing simple elongation of peptide chain and construction of hybrid hydrazino peptidomimetics with either alternating (like in **10**) or sequential (like in **11**) distribution of hydrazino acids along the sequence.

Although hydrazino peptides can be considered as extension of the β-peptide concept, conformational preferences of hydrazino peptides are considerably less described. Acherar et al. (2013) studied mixed foldamers, and found various conformations of hybrid oligomers composed of α-amino and α-hydrazino acids. We assume that such conformational flexibility could be important for the adaptability of hydrazino peptides in interaction with biomolecules, particularly nucleic acids. It is known that minor groove of the DNA is target of many non-covalent binding compounds, where beside electrostatic and van der Waals interactions, hydrogen bonding pattern is essential for a recognition process (Lauria et al. 2007). Therefore, we plan to expand here described concept of peptidomimetics with alternating and sequential distribution of hydrazino units on series of derivatives, to probe the binding to nucleic acids.

## Conclusions

We have prepared *N*^α^-benzyl hydrazino acids (*N*^α^-Bn hAaa) and unprotected hydrazino acids (hAaa) and tested their utility in synthesis of di- and tripeptides. We found that the coupling to the activated *N*^α^-protected hydrazino acids depends on steric and conformational characteristics of nucleophile, while deprotection of benzyl moiety requires harsh reaction conditions. Contrary to that, unprotected hydrazino acids allowed fast and simple construction of hybrid peptidomimetics with alternating and sequential distribution of hydrazino units.

## Additional files

**Additional file 1: Figure S1.** 1H NMR spectra of compounds** 2c** and** 2e** ([D7]DMF)**2c**.
**Additional file 2: Figure S2.** Temperature-dependent 1H NMR spectra of** 2e** ([D7]DMF).

## Abbreviations

BOP: (benzotriazol-1-yloxy)*tris*(dimethylamino)phosphonium hexafluorophosphate
DCC: *N,N′*-dicyclohexylcarbodiimide
EDC: 3-(ethyliminomethyleneamino)-*N*,*N*-dimethylpropan-1-amine
HATU: 1-[*bis*(dimethylamino)methylene]-1H-1,2,3-triazolo[4,5-b]pyridinium 3-oxid hexafluorophosphate
HOBt: 1-hydroxybenzotriazole
HOSu: *N*-hydroxysuccinimide
NMM: *N*-methylmorpholine
PyBOP: (benzotriazol-1-yloxy)tripyrrolidinophosphonium hexafluorophosphate
TFA: trifluoroacetic acid
TLC: thin-layer chromatography
TMS: tetramethylsilane
