# Supplementary material for: Synthesis of hybrid hydrazino peptides: protected vs unprotected chiral α-hydrazino acids
Source: Springerplus. 2015 Sep 17;4:507. doi: 10.1186/s40064-015-1288-9 (PMC4573739; doi:10.1186/s40064-015-1288-9)
Supplement: Supplementary file 2 — Additional file 2: Figure S2. Temperature-dependent 1H NMR spectra of 2e ([D7]DMF). [file 40064_2015_1288_MOESM2_ESM.doc]

Supportinf Information

Synthesis of hybrid hydrazino peptides: Protected vs unprotected chiral α-hydrazino acids

Josipa Suć and Ivanka Jerić*

Division of Organic Chemistry and Biochemistry, Ruđer Bošković Institute

Bijenička cesta 54, 10000 Zagreb, Croatia

E-mail: [ijeric@irb.hr](mailto:ijeric@irb.hr)

Figure S2. Temperature-dependent ^1^H NMR spectra of **2e** ([D_7_]DMF)
